# Spatiotemporal brain network analysis of healthy humans based on magnetoencephalography and functional MRI in the resting state

**DOI:** 10.1186/1471-2202-16-S1-P155

**Published:** 2015-12-18

**Authors:** Margaret Y Mahan, Arthur C Leuthold, Apostolos P Georgopoulos

**Affiliations:** 1 Graduate Program in Biomedical Informatics and Computational Biology, University of Minnesota, Minneapolis, MN 55455, USA; 2 Brain Sciences Center, VA Health Care System, Minneapolis, MN 55417, USA; 3 Department of Neuroscience, University of Minnesota, Minneapolis, MN 55455, USA

## 

Modern graph theory provides powerful quantitative tools and measures for the analysis of complex networks. The network measures are critical for creating metrics that allow for the comparison of connectivity patterns and neural interactions across subjects and brain measurements. In addition, these measures provide a framework for the characterization of network structure by identifying local contributions of individual nodes and connections, as well as the network's global capacity to integrate information. Since neural communication is the basis of brain function, assessing the dynamic status of the brain as a network is important. However, a major challenge is how to reliably measure and assess the network, and evaluate its utility. For that purpose, we used resting-state magnetoencephalography (MEG) and functional magnetic resonance imaging (fMRI) recordings from 65 cognitively healthy women to reliably assess the brain as a dynamic network.

MEG time series were prewhitened using ARIMA(50,1,1) and fMRI time series were prewhitened using ARIMA(15,1,1) to yield practically white noise innovations. For MEG, nodes were defined to be the individual sensors (n = 248); for fMRI, a modified functional template [1] was used to define nodes equaling those used for MEG in order to compare networks across the two modalities. Connectivity matrices were then calculated using pairwise Pearson correlations between the processed (1) whole time series, and (2) windowed time series (fMRI = 6 s window, MEG = 50 ms window) of all nodes. Weighted graphs of each of these connectivity matrices were evaluated using the following metrics: (a) local connectivity (strength, diversity), (b) global connectivity (mean correlation coefficient), (c) local network properties (degree, clustering coefficient, local efficiency, betweenness centrality), and (d) global network properties (smallworldness, centrality, global efficiency, path length, assortativity, transivity).

For the whole series, this analysis yielded MEG and fMRI "static" networks for each subject, characterized by the set of the values obtained for the parameters above. The effect of age was assessed by performing a multivariate regression where the network parameters were the dependent variables and age was the independent variable. Through an iterative, stepwise procedure those parameters that varied systematically with age were detected and retained. This analysis was performed for both the MEG and fMRI data, and the resulting MEG- and fMRI-based network parameters were compared. Similarly, for the windowed series, the same analyses were performed per time window to yield a "dynamic" network variation and the variation/stability of network structure over time was assessed. This provided an additional temporal network measure for evaluating the effect of age on brain network structure. We believe that this approach is at the heart of assessing brain networks at varying time scales, at different states in health and disease, and during performance of different tasks.

## References

[B1] Power JD Cohen AL Nelson SM Wig GS Barnes KA Church JA Vogel AC Laumann TO Miezin FM Schlaggar BL Petersen SE Functional network organization of the human brain Neuron 2011 72 665 678 22099467 10.1016/j.neuron.2011.09.006 PMC3222858

